# Revealing Contamination and Sequence of Overlapping Fingerprints by Unsupervised Treatment of a Hyperspectral Secondary Ion Mass Spectrometry Dataset

**DOI:** 10.1021/acs.analchem.1c01981

**Published:** 2021-10-13

**Authors:** Nunzio Tuccitto, Alessandra Bombace, Alessandro Auditore, Andrea Valenti, Alberto Torrisi, Giacomo Capizzi, Antonino Licciardello

**Affiliations:** † Consorzio per lo Sviluppo dei Sistemi a Grande Interfase, CSGI, Viale A. Doria 6, 95125 Catania, Italy; ‡ Department of Chemical Sciences, Università degli Studi di Catania, Viale A. Doria 6, 95125 Catania, Italy; § Electrical, Electronic and Computer Engineering, Università degli Studi di Catania, Viale A. Doria 6, 95125 Catania, Italy

## Abstract

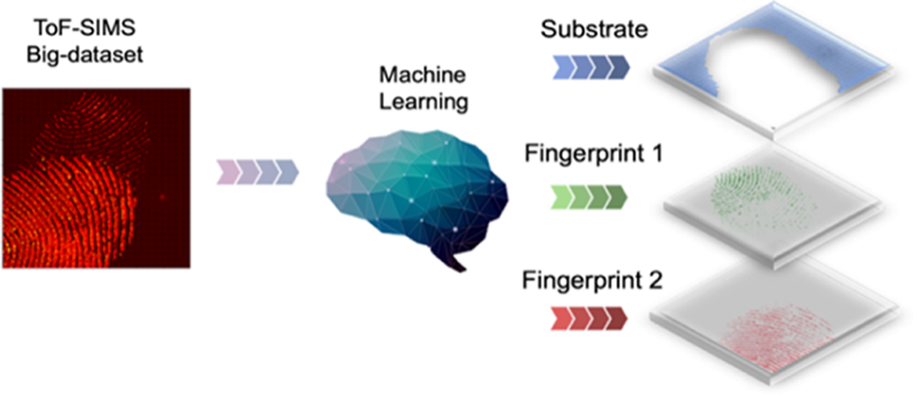

Time-of-flight secondary ion mass spectrometry (ToF-SIMS) has been successfully applied for chemical imaging of overlapping fingermarks. The resulting big dataset has been treated by means of an unsupervised machine learning approach based on uniform manifold approximation and projection. The hyperspectral matrix was composed of 49 million pixels associated with 518 peaks. However, the single-pixel spectrum results in a very poor signal intensity, mostly like a barcode. Contrary to what has been reported in the literature recently, we have not applied a crude approach based on binning but a sophisticated machine learning method capable of separating the chemical signals of the two fingerprints from each other and from the substrate in which they were impressed. Moreover, using ToF-SIMS, an extremely surface-sensitive technique, the sequence of deposition of the fingerprints has been determined.

## Introduction

Fingerprints are a unique defined pattern of dermal physical secretion developed from the friction of finger crests on surfaces. The biometric pattern on the finger skin is produced by minuscule conical papillae, in which thin crests are divided by subtle furrows. Crests leave curves, bows, and vortices on fingers. Distinctive dimensions and relative distances are unique to each human finger, making them individualistic anatomic features. Fingerprints are categorized as visible and invisible. Visible fingerprints are formed when the skin has traces of colored substances such as blood, ink, pollutants, or other chemicals, and invisible, when no such substances are found, and specific treatments are required to reveal them. Time-of-flight secondary ion mass spectrometry (ToF-SIMS) chemical imaging is a field of increasing interest owing to the enormous quantity of forensic information it can generate.^1−4^ ToF-SIMS has been successfully applied to chemical imaging of banknotes for (a) revealing invisible fingerprints,^5^ (b) identifying illicit drugs on fingerprints,^6^ (c) age-dating of fingerprints based on the diffusion of organic molecules,^7^ and (d) chronicling fingerprint deposition on documents.^8,9^

This study presents a new strategy to deal with vast datasets and training binned datasets through a neural network. We used the uniform manifold approximation and projection (UMAP) algorithm to carry out the dimension reduction technique. However, the approach is generic in purpose and can be undertaken through other algorithms such as PCA,^13−15^ t-SNE,^16^ and self-organizing maps.^10^

## Materials and Methods

Two fingerprints from two volunteers were impressed partially overlapping on a clean glass surface. Without any further sample treatment, high-resolution, static ToF-SIMS images were acquired with a ToF-SIMS IV (ION-ToF) instrument using a Bi_3_^+^ analysis beam (bunched mode, 25 keV, ∼0.1 pA, 256 × 256 pixels, single scan, rastered over 500 μm × 500 μm). The primary ion fluence was kept below 1 × 10^10^ ions × cm^–2^, a value far below the static conditions. As the area to be analyzed exceeded the maximum raster size of the primary ion beam, the acquisition of a chemical image of both fingerprints required the macroraster mode feature of the instrument to be exploited, collecting 30 × 30 stacked single raster scans; thus, a 15 × 15 mm^2^ area was investigated. To perform the data treatment, image datasets were exported from SurfaceLab 6.5 software (ION-ToF GmbH) to the general raw data format. They were imported into Python 3.8-based scripts developed in lab and run on a workstation equipped with Intel 16 cores i9 9900K processors and 256 GB of RAM. We imported Numpy as the linear algebra library, Matplotlib for data plotting, PIL for image editing, and UMAP-learn for uniform manifold approximation and projection. Please refer to Supporting Information for the pseudocode.

## Results and Discussion

**Figure 1:**
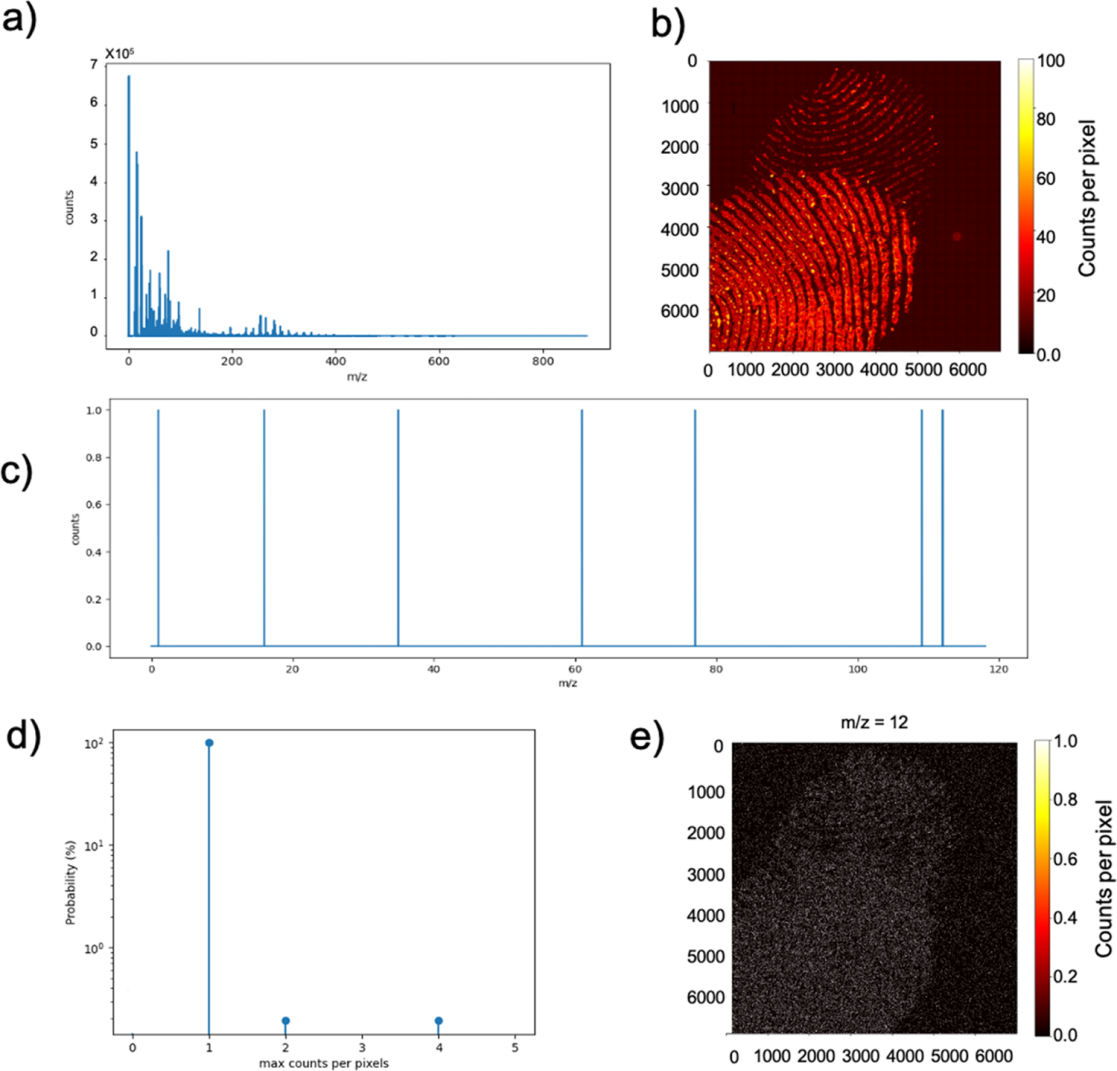
Partial overlapping of two fingerprints/maximum TIC per single pixel.

Figure 1a shows the secondary ions mass spectrum acquired from the entire analyzed area. Significant peaks at *m*/*z* values under approximately *m*/*z* 400 are observed. A peak list has been built by 518 peaks identified in the mass spectrum. Figure 1b shows the total ion map of the 7000 × 7000 (49 million) pixel macroraster images. Hence, a multispectral analysis of the raw file facilitates the construction of a huge matrix dataset, composed of 49 million × 518 elements.

As shown in Figure 1b, the maximum total ion count (TIC) per single pixel of all integrated 518 sectors does not exceed 100 counts (as it can easily be deduced from the color bar in Figure 1b). Considering that the TIC map is the sum of all the signals acquired by the detector, comprising hundreds of peaks (as shown in Figure 1a), the image represents partial overlapping of two fingerprints. However, information related to individual signals within the dataset is very poor, clearly visible from the mass spectrum reconstructed from a single pixel reported and contains only single-count signals, as revealed in Figure 1c. This consideration can be extended to the entire image because the outcome of the statistical analysis reported in Figure 1d represents the probability plot of the maximum detected counts based on the integration of all 518 peaks, in which the maximum number of counts per single pixel is equal to one. Despite the huge size of the raw data, the results of the statistical data are poor. Figure 1e shows the false-color map created by integrating the signals from the peak at *m*/*z* 12, assigned to carbon. Despite being one of the most intense peaks in the total ion spectrum (refer to Figure 1a), only a black-and-white image is obtained because the counts in each pixel are either null (black color) or unitary (white color). This outcome is common with ToF-SIMS macroraster images or with 3D images obtained by depth profiling of organic-based samples. In both instances, reducing the acquisition time is crucial to avoid ion bombardment-induced damage accumulation phenomena used for maintaining the ion dose below the so-called “static limit” and keeping the analysis duration controlled.

Consequently, the operator is forced to make time-conservative choices that often generate difficulties in the ex-post-investigation of the acquired data. A possible solution is to apply the analysis conditions to enhance the secondary ions’ yield. Studies have reported some examples of successful approaches that include making use of reactive gases,^17^ metal nanoparticles,^18^ and primary reactive beams.^19^ However, these protocols are inapplicable because they do not always give satisfactory results. Their effectiveness often depends on the chemical nature of the sample under investigation. Binning neighboring pixels is a procedure commonly applied to ToF-SIMS images with a low signal-to-noise ratio, which generates images with a statistically significant signal.

**Figure 2:**
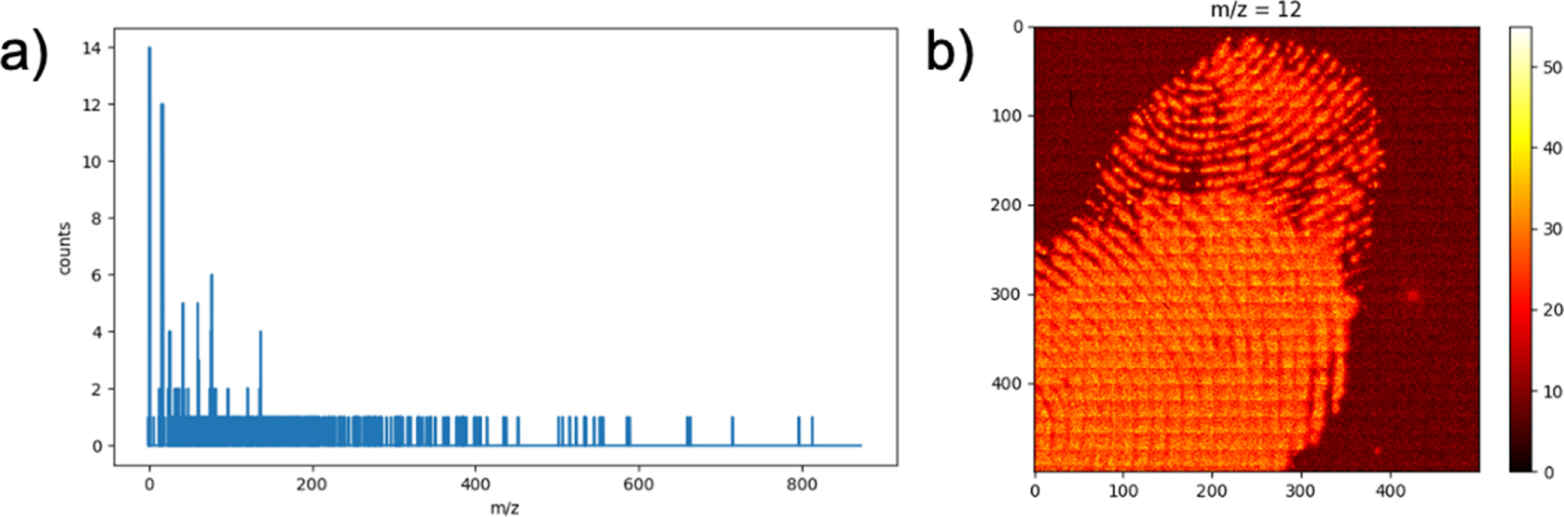
Spectrum related to binning of a 10 × 10 neighboring pixels/false-color image relative to the integration of the peak assigned to the C-ion.

Figure 2a shows the spectrum related to binning 10 × 10 neighboring pixels. The spectrum is now richer in counts and far more intelligible than the one shown in Figure 1c, which resembles an unintelligible barcode more. Figure 2b shows the false-color image relative to the integration of the peak assigned to the C-ion having the centroid at *m*/*z* = 12. Compared with the original nonbinned image (see Figure 1d), this image reveals a greater intensity, giving more statically relevant information. Therefore, the binning produces less massive multispectral data matrices, but it is statistically richer and more useful for data processing. However, pixel binning leads to a deterioration of lateral resolution, causing a loss of spatial information in the chemical image. This is relevant in contemporary forensic applications because analyzing even small features of fingerprint ridges has become a vital case.^20^

**Scheme 1:**
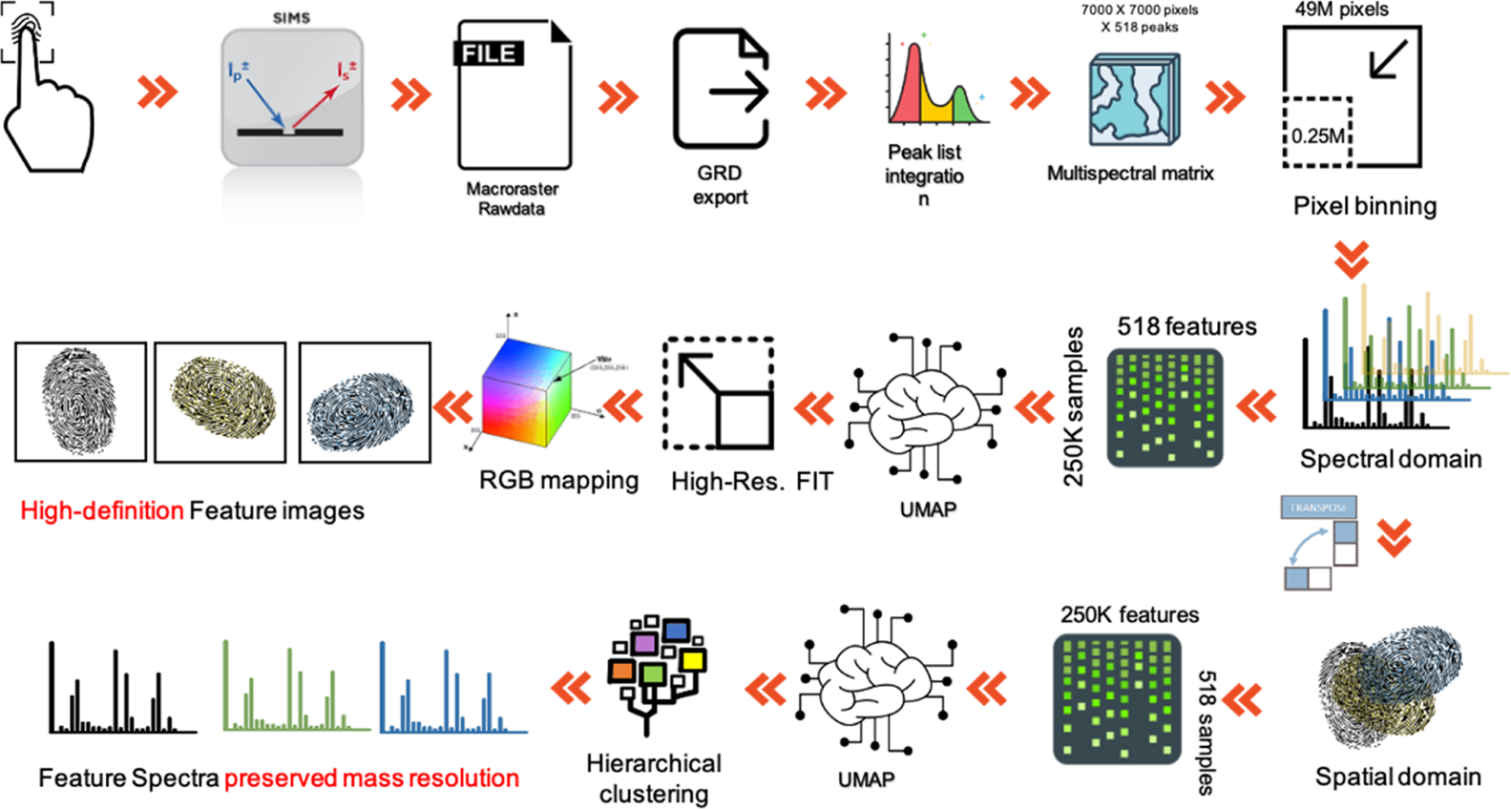
Learning Clustering Data from the Binned Dataset Using the Neural Network

Our strategy is based on the machine learning methodologies, in which a neural network learns the necessary information from the binned data. Then, the trained neural network retrofits information on the nonbinned dataset. The rationale is based on the following criterion: since the raw data to be used for the neural network training are statistically poor to be processed for reliable information, the pixel binning process enables the neural network to learn datasets statistically richer, enough to recognize and cluster the original nonbinned data in a post-training phase without sacrificing lateral resolution. In a nutshell, the neural network learns how to cluster data from the binned dataset, enabling cluster assignment application of the nonbinned data. The procedure is schematized in Scheme 1.

We analyzed the sample by acquiring a macroraster ToF-SIMS image. The image raw data were exported into a readable format using the nonproprietary vendor software (nominally GRD). The mass spectrum of the entire image was reconstructed (Figure 1a), and a peak list containing 518 peaks was defined. The integration of the signal in the peak list leads to a hyperspectral array. Each of the 518 maps was binned by summing the data to 14 × 14 neighboring pixels, reducing the size of the hyperspectral images from 49 million pixels to 250 thousand pixels. The binned matrix was subjected to a dimensional reduction in the mass domain (or ToF) after transposition of the spatial domain (or pixels). We used the parametric UMAP manifold learning technique for dimension reduction,^21,22^ recently applied to nonlinear dimension reduction of hyperspectral data gathered from mass spectrometry techniques that comprised ToF-SIMS.^11^ A study provides a detailed description of the underlying mathematics related to UMAP.^23^ In the process, the algorithm first constructs a fuzzy topological representation of the dataset. Then, it optimizes the low-dimensional representation to have a close possible fuzzy topological representation, measured by cross-entropy. Although UMAP is grounded in a complex mathematical foundation, a brief mathematical description is provided to understand the working process of this algorithm. The UMAP computation technique consists of two processes: first, it constructs a graph of local relationships between objects (pixels or ToF) in datasets. Then, it optimizes embedding in a low-dimensional space to preserve the graph structure. The parametric UMAP approach performs the second step through parameter optimization based on a deep neural network. We applied a reduction in the mass domain up to three embeddings to compute a graphical representation of the binned dataset. Subsequently, the trained neural network was used to backfit the nonbinned dataset, which learned a set of weights that preserved the structure of the graph related to the binned dataset. According to RGB mapping, color scale mapping resulted in high-resolution similarity maps. Only two embeddings were necessary for a reduction in the spatial domain. Using a hierarchical clustering algorithm, it was possible to map, in false colors, the peaks belonging to each cluster. Similarly, the trained neural network helped to reconstruct the characteristic spectra of each cluster, in which the peak intensity is calculated as a product of the percentage belonging to the cluster, identified by the hierarchical algorithm and the counts of the nonbinned dataset. However, large-area analyses using ToF-SIMS are a time-consuming process. For instance, the acquisition of the image required approximately 2.5 h. Although the signal-to-noise ratio could be improved by the accumulation of several scans without exceeding the static limit, this approach has a huge impact on the time analysis time. This solution is unfavorable because of the higher costs and possible adverse effects owing to the possible instabilities of certain instrumental parameters. For this reason, we carry out a single scan, keeping the acquisition time as low as possible. The approach aims to check if the proposed methodology is suitable to extract chemical information from ToF-SIMS images characterized with a very low signal-to-noise ratio, without loss of lateral resolution. The time to carry out the data processing is lower in a dedicated computing workstation. However, performing long acquisitions with the ToF-SIMS instrument in macroraster mode has implications on the stability, helping to avoid the related running costs. Both limitations can be solved using the proposed method.^12^

**Figure 3:**
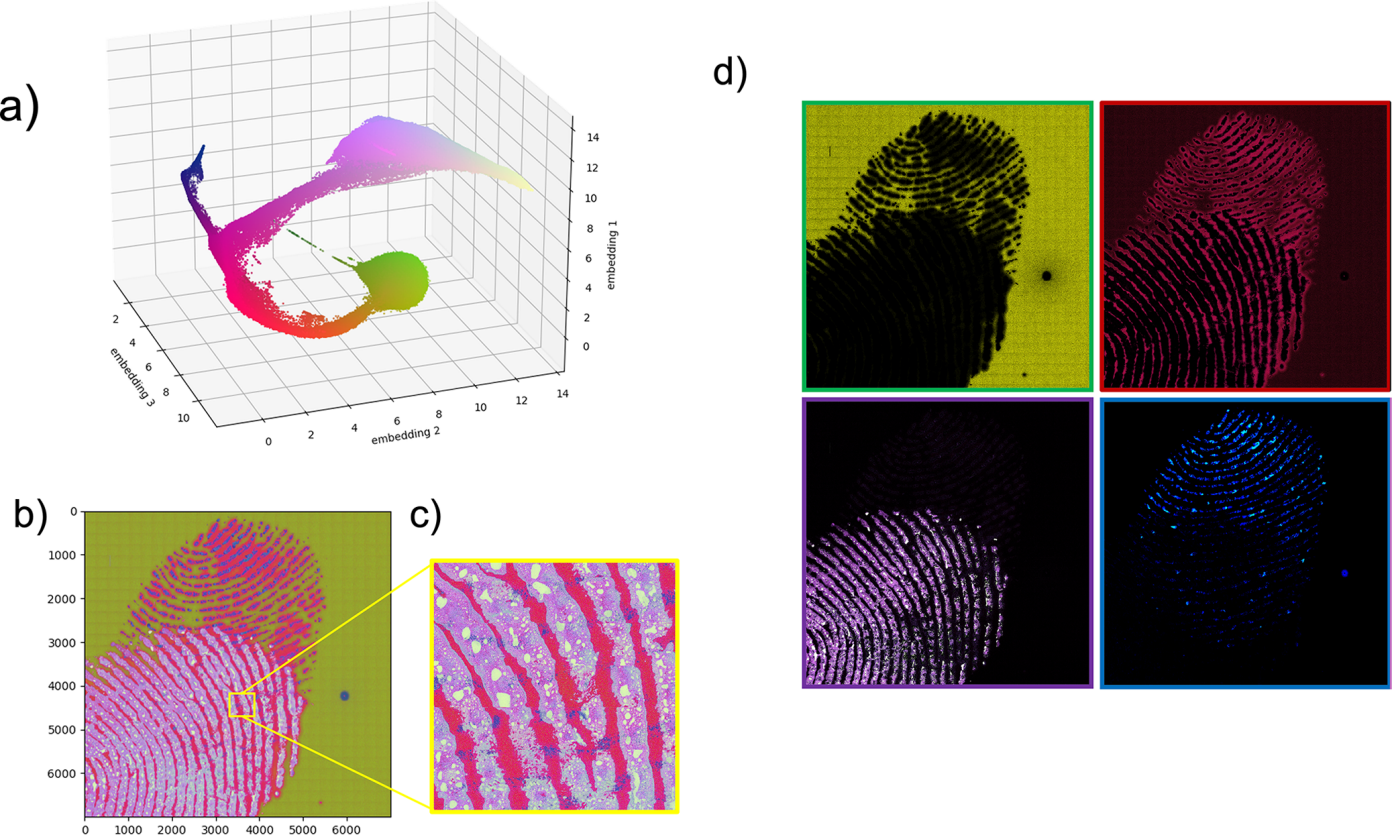
3D projection of the 49 million pixels from UMAP embedding.

Figure 3a shows a 3D projection of the 49 million pixels from UMAP embedding. From the analysis, 3D is adequate to identify four clusters. In addition, RGB mapping helps to identify different regions in the plot and enables individual pixel color assignment. Three clusters are well separated (green, blue, and light pink), all joined to a fourth red cluster. Since the index of each pixel in the dataset is known, reconstructing similarity maps in false colors is possible. Figure 3b shows the reconstruction of the nonbinned dataset. The outcome shows that the proposed procedure facilitates recognition and distinction of two fingerprints through visualization of discrete chemical features (revealed in blue and light-pink color) on a substrate (green color). In our proposed procedure, the lateral resolution is preserved, which facilitates detailed inspection of complex portions of the sample, especially in areas where the two fingerprints overlap—as shown in the magnified portion in Figure 3c. In forensic applications, retaining high resolution may lead to a significant difference between “guilty or not guilty”. By adequately separating the color channels, details of the four clusters are enhanced, as shown in Figure 3d. Note that the fingerprint assigned to blue is unequivocally placed under the other assigned to light pink. The pixels clustered and represented by red are related to areas belonging to both fingerprints with similar chemical compositions. However, the chemical information is obtained from the analysis of the transposed dataset.

**Figure 4:**
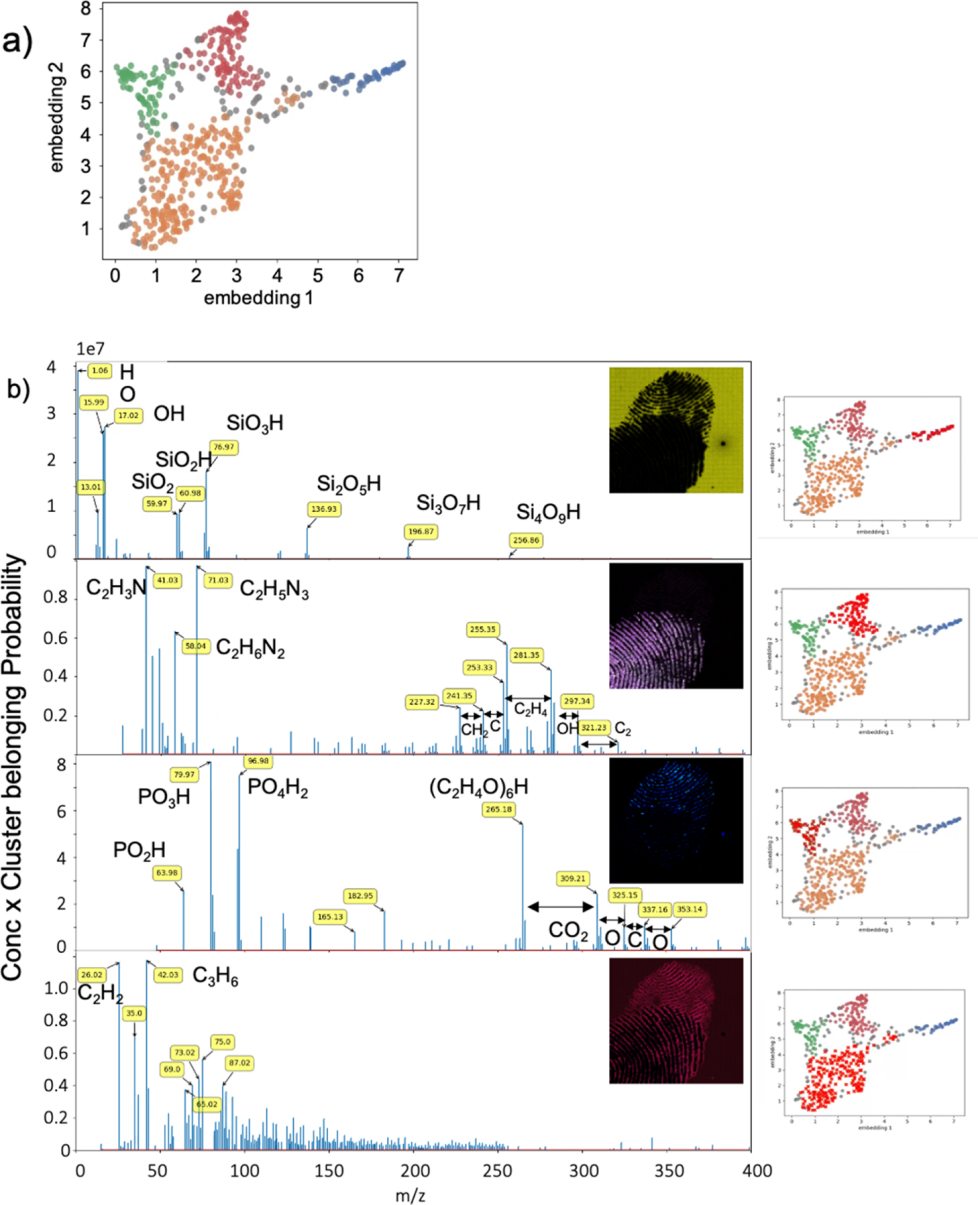
Two-dimensional embedding of the processed dataset after matrix transposition.

Figure 4a shows the two-dimensional embedding of the processed dataset after matrix transposition. The 518 dimensions were subjected to clustering and plotted in colors belonging to the four clusters (plus the gray color assigned to the noisy points not clustered by the algorithm). Figure 4b shows the mass spectra relative to each cluster and the corresponding false-color map. The tentative peak assignment confirms that the first cluster is relative to the mass spectrum of the substrate (i.e., silicon oxide-based glass). The fingerprint associated with the light-pink cluster has low mass peak characteristics in correlation with the nitrogen-containing organic fragments and unidentified clusters at high masses (between *m*/*z* 250 and 350). The outcome has differences in masses in correlation with smaller organic fragments. The blue-colored fingerprint has a peculiar mass spectrum, characterized by the peaks assigned to phosphate compounds at *m*/*z* below 100 and oligomer fragments of ethylene oxide-based compounds, normally used in cosmetics. The red clustered zone is characterized by a spectrum rich in peaks at high masses and a lower number of peaks attributed to hydrocarbon fragments at low masses. This confirms that these are not strongly discriminating zones between the two imprints. As for lateral resolution, maintaining high resolution in mass is vital for the recognition of substances of forensic interest. A database comparative analysis or a more rigorous assessment of the characteristic fragments of the various clusters would generate more information regarding the origin of these chemical features, with important forensic repercussions. Such a study, which is underway in our laboratory, is beyond the scope of this paper. However, the data revealed so far demonstrate that the big-data-processing procedure applies in an unsupervised mode to datasets derived from ToF-SIMS analysis with a high mass resolution and pixel definition of tens of megapixels. This is the only choice that needs to be made regarding binning between neighboring pixels.

**Figure 5:**
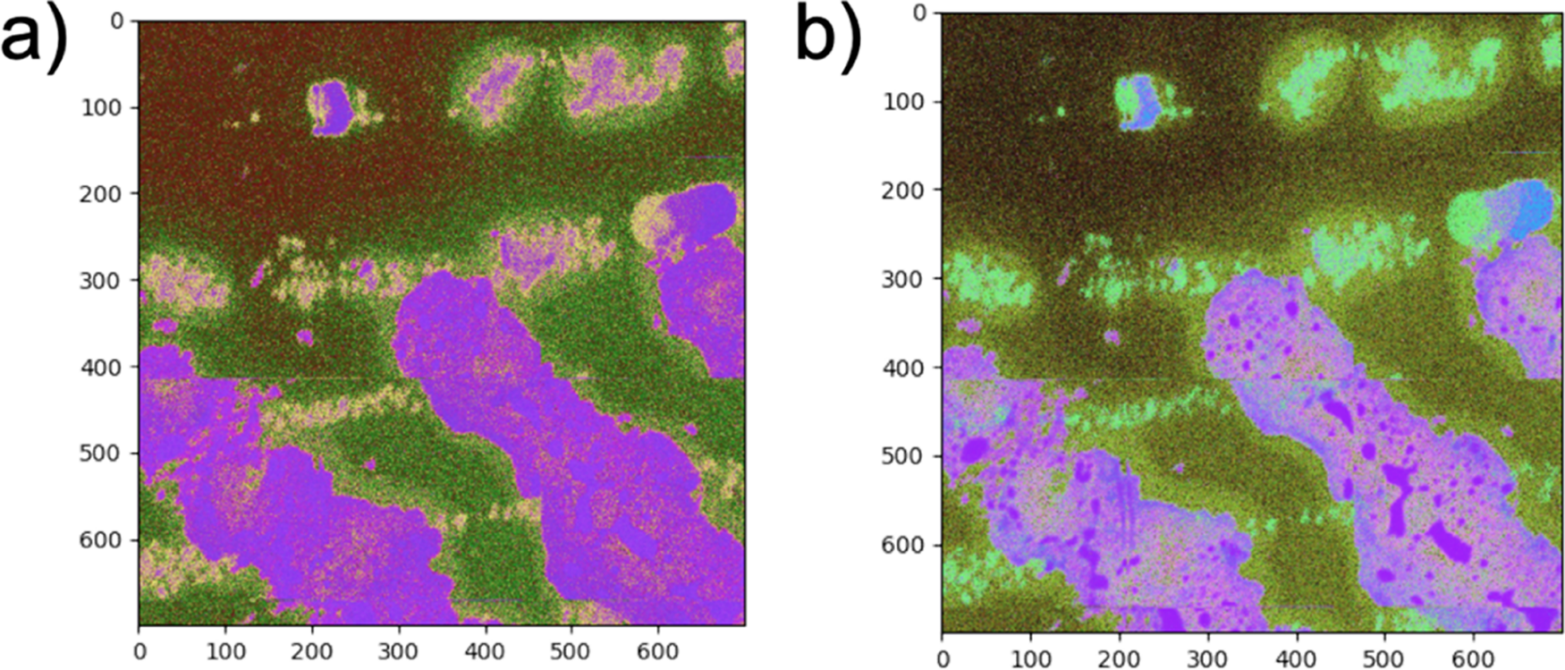
Comparison of the proposed method performed on the same fingerprint dataset subset with neural networks trained using a differing binning level.

Figure 5 compares the proposed method performed on the same subset of fingerprint datasets with neural networks trained using a differing binning level. The results show that excessive compression of the initial data leads to a loss of the information needed to discriminate between fingerprints. From Figure 5a, by performing a strong binning between nearby pixels, the neural network can discriminate the substrate pixels from those of the fingerprints, but it cannot distinguish between them. Please note that in Figure 5a, both fingerprints have the same color (pink), indicating that they are similar. The results of coarser binning in a dataset are unsuitable for appropriate training of the network because the initial data are statistically poor. From Figure 5, by decreasing the binning to 4 × 4, the distribution of the dots in the 3D embedding plot becomes highly scattered, and the substrate cannot be easily distinguished from one of the two fingerprints. One of the fingermarks is associated with the same green color of the substrate, albeit still visible by employing the intensity contrast. Therefore, the best condition is the intermediate binning value of approximately 14 × 14, which reduces the 7000 × 7000 of the entire hyperspectral images into 500 × 500—see Figure S1 for a more exhaustive report on the effect of pixel binning. This value is used throughout the study. Thus, such a value is sample-dependent, determined for any dataset. We are still studying how to achieve this unique protocol, but the procedure is rendered unsupervised, and the user can judiciously choose the best level of binning similar to compression methodology.

## Conclusions

In this paper we have described a very promising neural network-based routine that enables extraction of relevant information from big datasets obtained from ToF-SIMS imaging of large areas with good lateral resolution but very poor signal-to-noise ratio. We have shown that it is possible to overcome the loss of lateral resolution, intrinsic in the commonly used binning process, by using a neural network-based routine. The method involves the training of the neural network by using a binned dataset that enables improvement in the signal-to-noise ratio of the chemical images even with loss of lateral resolution. The same neural network has been applied back to the original nonbinned dataset, gaining high lateral resolution image data again. We have applied our method to samples that could be of relevance to forensic science, such as fingerprint detection, analysis of chemical compounds transferred together with fingerprints, and fingerprint impression sequence. We are confident that it can be successfully applied to other contexts such as 3D imaging analysis of biological samples in which the signal-to-noise ratio is usually low because of the limited number of primary ion scans allowed during analysis to keep the modifications of molecular structures, caused by ion-induced damage, as low as possible.

## Supporting Information Available

Further false-color images and related UMAP embeddings, as calculated by trained neural networks by using several binned datasets of a portion of the dataset and the simplified Python pseudocode (PDF)
