# Supplementary material for: Revealing Contamination and Sequence of Overlapping Fingerprints by Unsupervised Treatment of a Hyperspectral Secondary Ion Mass Spectrometry Dataset
Source: Anal Chem. 2021 Oct 13;93(42):14099–105. doi: 10.1021/acs.analchem.1c01981 (PMC8552212; doi:10.1021/acs.analchem.1c01981)
Supplement: Supplementary file 1 — ac1c01981_si_001.pdf [file ac1c01981_si_001.pdf]

Supplementary Information

Revealing Contamination and Sequence of Overlapped Fingerprints by Unsupervised Treatment of Hyperspectral Secondary Ion Mass Spectrometry Dataset

Nunzio Tuccitto,[a,b,]* *Alessandra Bombace,*[b] *Alessandro Auditore,*[b] *Andrea Valenti,*[a] *Alberto Torrisi,*[b] *Giacomo Capizzi,*[c] *and Antonino Licciardello*[a,b,*]

AUTHOR ADDRESS

a) Consorzio per lo Sviluppo dei Sistemi a Grande Interfase, CSGI, Viale A. Doria 6, 95125 Catania, Italy

b) Department of Chemical Sciences, Università degli Studi di Catania, Viale A. Doria 6, 95125 Catania, Italy.

b) Department of Chemical Sciences, Università degli Studi di Catania, Viale A. Doria 6, 95125 Catania, Italy.

c) Electrical, Electronic and Computer Engineering, Università degli Studi di Catania, Viale A. Doria 6, 95125 Catania, Italy.

*Correspondence to: nunzio.tuccitto@unict.it, alicciardello@unict.it

Table of content

- Additional figure
- Simplified Pseudo Python Code:

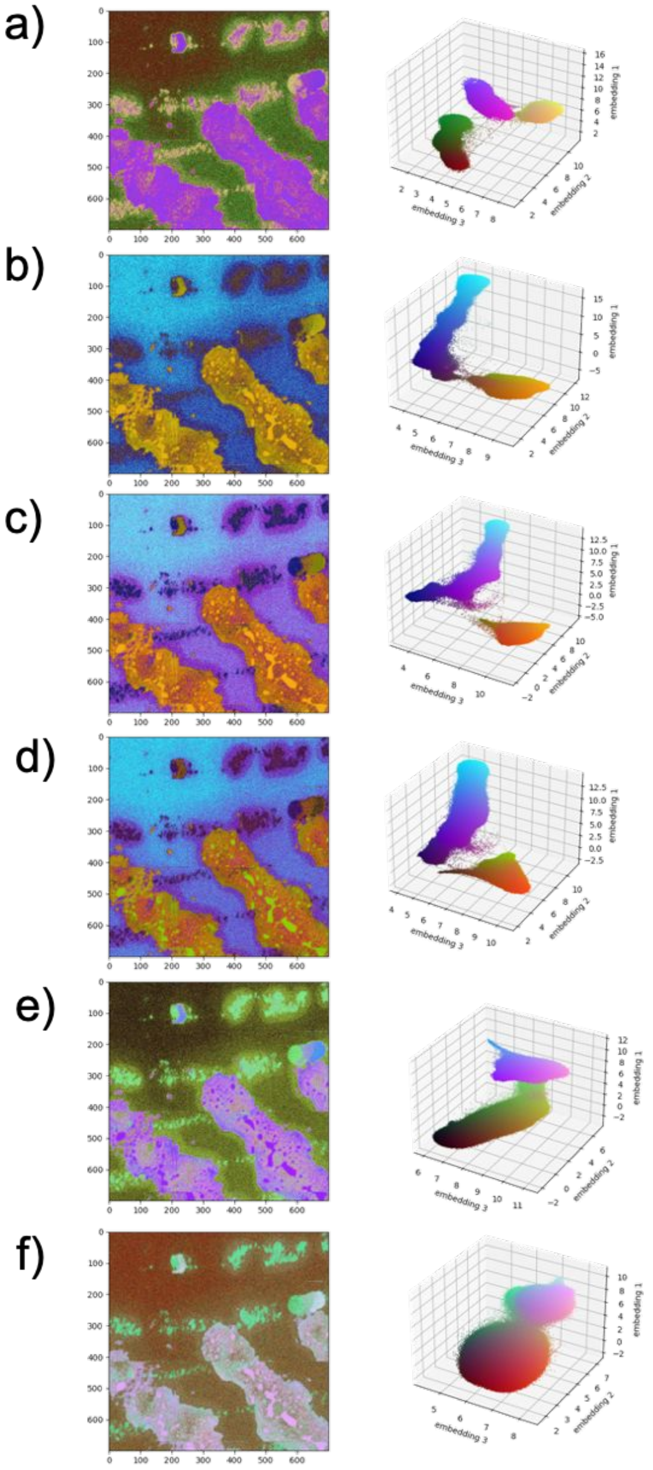

- Figure S1 False color image of a portion of the non-binned dataset coloring each pixel according to a RGB mapping of the 3 embeddings scaled normalizing between 0 and 255 and the relative three-dimensional projection of the 3 UMAP embeddings as calculated by trained neural network by using several binned datasets: a) 70x70, b) 35x35, c) 14x14, d) 7x7, e) 4X4, f) 2X2.

Simplified Pseudo Python Code:

```
import umap
import numpy as np

def rebin(arr, new_shape):
    """Rebin 2D array arr to shape new_shape by averaging."""
    shape = (new_shape[0], arr.shape[0] // new_shape[0],
            new_shape[1], arr.shape[1] // new_shape[1])
    return arr.reshape(shape).mean(-1).mean(1)

 """load dataset"""
x = np.load('foo.npy').astype(np.uint8)
x = x.reshape(7000,7000,518)

"""binning dataset"""
binning_to = int(700) #binning factor
y = np.zeros((binning_to,binning_to,518))
for i in range (518):
    print (i)
    y[:,:,i]=rebin(x[:,:,i], (binning_to,binning_to))
y = y.reshape(binning_to**2,518)

"""learn on binned dataset"""
mapper = umap.UMAP(n_neighbors = 15, n_components=3, low_memory=True, metric='cosine',init = 'spectral', spread =
1.0, min_dist = 0.1, verbose = 'true').fit(y)


"""transform NON binned dataset"""
x = x.reshape(7000*7000,518)
transformed = np.zeros((7000**2,3))
for i in range(0,1000*7000,100000):
      transformed[start*7000+i:start*7000+i+100000,:] = mapper.transform(x[i:i+100000,:])
```
